# Resection of intradural intramedullary subependymoma of the cervicothoracic spine: 2-dimensional operative video

**DOI:** 10.3171/2023.6.FOCVID2381

**Published:** 2023-10-01

**Authors:** Joshua Vignolles-Jeong, Maxwell D. Gruber, Vikas Munjal, Santino Cua, Justin Baum, Vikram B. Chakravarthy

**Affiliations:** Department of Neurological Surgery, The Ohio State University, Columbus, Ohio

**Keywords:** subependymoma, spine, intradural intramedullary

## Abstract

This video presents the case of a 44-year-old male with a 2-year history of pain in the left upper extremity that had worsened over the course of the last 6 months with associated weakened grip strength and had extended into his right arm. T2-weighted sagittal and axial MRI demonstrated an expansive nonenhancing solid intramedullary lesion extending from C5 to T1. The patient underwent a C5–T1 laminectomy and laminoplasty with near-complete resection of the intradural intramedullary subependymoma. At 3 months’ follow-up, he reported doing well and had experienced significant improvement in motor strength with ongoing therapies.

## Transcript

We are presenting the case of a resection of an intradural intramedullary subependymoma of the cervicothoracic spine, a 2-dimensional operative video.

### 0:32 Case History.

We present the case of a 44-year-old male with a past pertinent medical history of pain of 2 years’ duration in the left upper extremity in a C7–T1 distribution. The patient reported that the pain had worsened over the course of the past 6 months with spread and extension into his right arm. Additionally, he reported weakened grip strength in his left hand. The patient denied any bowel or bladder complaints, bilateral lower-extremity weakness, and/or gait abnormalities, and/or ataxia. On physical examination, the patient was 5 out of 5 strength bilaterally in the upper extremities with all pertinent muscle groups tested. Decreased sensation was noted in the left C7, C8, and T1 distributions. Reflexes were 2+ throughout and no Hoffman sign was appreciated bilaterally.

### 1:16 Preoperative Imaging.

On review of imaging, T2-weighted sagittal and axial MRI images demonstrated an expansive nonenhancing solid intramedullary lesion extending from C5 to T1. In an effort to gain multiple insights into pathology and optimal treatment management strategies, the patient was discussed in a multidisciplinary spine tumor board. Additionally, with concern for an enlarging, expansile mass, the patient was taken to the operating room for biopsy prior to initiating resection. Frozen pathology and histology demonstrated subependymoma.

### 1:53 Rationale for the Procedure.

The risks and benefits were discussed extensively with the patient, and he consented to the procedure. Rationale for pursuing surgical intervention included progressive neurological symptoms with spread into the distal extremity and contralateral side, radiographic imaging demonstrating the expansile mass and concern for tumor growth, and overall morphology in size of the tumor.

### 2:15 Alternatives for Treatment.

Other alternatives were discussed with the patient. Alternatives included continued observation, surgical resection, biopsy plus-or-minus radiation, with fractionated photon or proton radiation being considered, as well as multisession radiosurgery.

### 2:31 Description of Setup.

For this procedure, the patient’s head needs to be stabilized and maintained with a Mayfield head holder in the prone position. Necessary equipment include neuromonitoring, including motor evoked potentials, somatosensory evoked potentials, and D-wave. Additional equipment necessary includes an ultrasonic aspirator and operative microscope. Key surgical steps include posterior approach with C5–T2 decompression, resection of the intradural intramedullary lesion, and laminoplasty.

### 3:02 Intraoperative Ultrasound.

During the case, intraoperative ultrasound was utilized to confirm the extent of the intradural tumor and define the extent of myelotomy and opening dural resection.

### 3:13 D-Wave Device Placement and Dural Opening.

We begin this case in the postoperative biopsy state. We start by carefully taking down the prior suture to the level of the dura. After carefully peeling back the layers of the dura, a D-wave electromonitoring device is placed first at the caudal end, followed by insertion at the cranial end. In opposition to motor evoked potentials or somatosensory evoked potentials, continuous neuromonitoring without concern for evoked potentials and temporary spastic transient movement was favored. As such, we used intradural D-wave monitoring to provide continuous feedback of neurological function.

The probe is carefully irrigated alongside its insertion to ensure adequate sliding and smooth entry. If resistance is felt, the D-wave is slowly pulled back and advanced in a different direction for a more favorable trajectory. The myelotomy site from prior biopsy is carefully incised, first caudally and extended cranially. In lieu of phase reversal for defining the midline raphe per Adelson et al.^6^ the midline was identified and incised at the convergence of the medullary vessels.

### 4:35 Tumor Segregation.

Next, carefully dissect the plane between normal spinal cord tissue and tumor. To fully define the dimensions of the tumor, we expanded the myelotomy caudally in a delicate fashion, again, following the midline raphe.

### 5:05 Tumor Debulking.

Once an adequate dissection plane has been created, the ultrasonic aspirator is brought to the field and the tumor is taken down layer by layer to minimize any pulling or retraction of the normal spinal cord.

Once adequate resection is performed with the ultrasonic aspirator, additional dissection is performed to liberate any additional tumor components from surrounding native tissue. This back and forth is continuously completed between sharp dissection, creating a plane between tumor and spinal cord and ultrasonic aspirator to minimize retraction to the spinal cord.

### 5:55 Ultrasound Confirmation of Tumor Resection.

Following tumor removal, intraoperative ultrasound was utilized to verify the parameters of the tumor bed. Once ultrasound and full visualization deemed resection of the tumor optimal, we opted to close the surgical incision. In this case, we preferred to close with the running 4-0 Nurolon suture for appropriate postoperative observation. Following closure, the wound bed is copiously irrigated.

### 6:33 Neuromonitoring Modalities.

When considering intraoperative use of neuromonitoring, there are various targets, mechanisms, pros, and cons to consider. Of note, in this case, we elected to utilize direct waves or D-waves. The thought process being greater, continuous feedback of motor potentials. This was contrasted against MEPs or motor evoked potentials and SSEPs (somatosensory evoked potentials), which are reliant upon a generated evoked motor potential for time point analysis of neurological integrity of the descending motor tracks and ascending somatosensory pathways.^1–5^

### 7:06 Direct Wave Monitoring.

In the case study presented herein, we opted to utilize D-wave monitoring in the intradural setting for a greater degree of sensitivity and specificity in monitoring lateral corticospinal tracts.

### 7:31 Three-Month Follow-Up.

At 3 months’ follow-up, the patient reported that he was doing well and was noticing significant improvement in motor strength with ongoing therapies. On neurological exam, the patient was noted to be grossly neurologically intact, albeit decreased strength in hip flexion, quadricep extension, and hamstring strength. The patient was noted to have negative clonus bilaterally with 2+ deep tendon reflexes throughout.

### 7:55 Follow-Up Imaging.

On follow-up imaging, T2-weighted sagittal and axial MRI sections demonstrated postoperative changes consistent with the C5–T1 laminectomy and laminoplasty with near-complete resection of the intramedullary subependymoma.

## Disclosures

The authors report no conflict of interest concerning the materials or methods used in this study or the findings specified in this publication.

## Author Contributions

Primary surgeon: Chakravarthy. Assistant surgeon: Baum. Editing and drafting the video and abstract: Vignolles-Jeong, Gruber, Munjal, Cua. Critically revising the work: Vignolles-Jeong, Gruber, Munjal, Cua, Chakravarthy. Reviewed submitted version of the work: Vignolles-Jeong, Gruber, Munjal, Cua, Chakravarthy. Approved the final version of the work on behalf of all authors: Vignolles-Jeong. Supervision: Chakravarthy.

## Supplemental Information

### Patient Informed Consent

The necessary patient informed consent was obtained in this study.
